# Dietary Supplementation of Haskap Berry (*Lonicera caerulea* L.) Anthocyanins and Probiotics Attenuate Dextran Sulfate Sodium-Induced Colitis: Evidence from an Experimental Animal Model

**DOI:** 10.3390/foods13131987

**Published:** 2024-06-24

**Authors:** K. V. Surangi Dharmawansa, Andrew W. Stadnyk, H. P. Vasantha Rupasinghe

**Affiliations:** 1 Department of Plant, Food, and Environmental Sciences, Faculty of Agriculture, Dalhousie University, Truro, NS B2N 5E3, Canada; 2 Departments of Microbiology & Immunology and Pediatrics, Faculty of Medicine, Dalhousie University, Halifax, NS B3H 4R2, Canada; 3 Department of Pathology, Faculty of Medicine, Dalhousie University, Halifax, NS B3H 4R2, Canada

**Keywords:** inflammation, colitis, blue honeysuckle, probiotics, flavonoids, microencapsulation, probiotics

## Abstract

Haskap berry (*Lonicera caerulea* L.) is a rich dietary source of anthocyanins with potent anti-inflammatory properties. In this study, isolated haskap berry anthocyanins were encapsulated in maltodextrin and inulin (3:1) by freeze-drying to improve stability and bioavailability. The structural properties of microcapsules, encapsulation yield, efficiency, recovery, and powder retention were evaluated. The microcapsules that exhibited the highest encapsulation efficiency (60%) and anthocyanin recovery (89%) were used in the dextran sulfate sodium (DSS)-induced acute colitis in mice. Thirty-five BALB/c male mice of seven weeks old were divided into seven dietary supplementation groups (*n* = 5) to receive either free anthocyanins, encapsulated anthocyanins (6.2 mg/day), or probiotics (1 × 10^9^ CFU/day) alone or as combinations of anthocyanin and probiotics. As observed by clinical data, free anthocyanin and probiotic supplementation significantly reduced the severity of colitis. The supplementary diets suppressed the DSS-induced elevation of serum inflammatory (interleukin (IL)-6 and tumor necrosis factor) and apoptosis markers (B-cell lymphoma 2 and Bcl-2-associated X protein) in mice colon tissues. The free anthocyanins and probiotics significantly reduced the serum IL-6 levels. In conclusion, the dietary supplementation of haskap berry anthocyanins and probiotics protects against DSS-induced colitis possibly by attenuating mucosal inflammation, and this combination has the potential as a health-promoting dietary supplement and nutraceutical.

## 1. Introduction

Inflammation is the body’s protective mechanism against infections, toxins, and in some cases, self-antigens [1]. Unremitting acute and chronic inflammation are highly health-debilitating and proven risk factors for developing cancer [2]. Inflammatory bowel diseases, Crohn’s disease, and ulcerative colitis, in patients who are nonresponsive to treatments, are risk factors for colitis-associated colorectal cancer (CRC) [3]. Once the inflammation leads to the chronic stage, it generates oxidative stress-induced DNA damage, which then activates tumor-promoting genes and inactivates tumor-suppressor genes, thus contributing to the development of a cascade of events of carcinogenesis [4]. Ulcerative colitis affects approximately 1 of every 250 people in North America, and people with ulcerative colitis have a 1.7-fold higher risk for CRC compared with the general population. Further, more than 20% of IBD patients develop CRC within 30 years of disease onset, and >50% of these will die from CRC [5]. Therefore, the attenuation of colitis, while having immediate beneficial consequences, can be considered as a means to reduce the prevalence of CRC.

The consumption of colorful fruits and vegetables rich in plant flavonoids such as anthocyanins has been intensively studied for anticancer and anti-inflammatory properties [6]. Among anthocyanins, cyanidin-3-*O*-glucoside (C3G) exhibits significantly strong antioxidant, anti-inflammatory, and anticancer properties [7]. The metabolism of C3G in the gastrointestinal tract could produce bioactive phenolic metabolites, such as protocatechuic acid (PCA), phloroglucinaldehyde (PGA), vanillic acid, and ferulic acid, which enhance C3G bioavailability and contribute to both the mucosal barrier and microbiota [8]. Tight junctions (TJs) consist of at least 40 different proteins, including claudins and occludins, located at the apical side, which play a vital role in contributing to the sealing of paracellular spaces of epithelium, restricting the invasion of inflammation causative agents [9]. It is noteworthy that anthocyanins improve the intestinal TJ barrier integrity by promoting the expression of crucial barrier-forming TJs such as occludin, claudin-5, and zonula occludin-1 [10]. Cytokines are important mediators of inflammation and elevated levels of major proinflammatory cytokines such as tumor necrosis factor (TNF), interleukin (IL)-6, IL-1β, and interferon-gamma (IFN-γ) are observed in patients with ulcerative colitis and also in chemically induced colitis models [11]. As has been observed, anthocyanin has the potential to prevent increases in proinflammatory cytokines potentially by suppressing the activation of nuclear factor-kappa B (NF-κB) and extracellular signal-regulated kinase (ERK) [12]. Emerging evidence suggests that haskap berry (*Lonicera caerulea*) is more potent than all the other types of berries due to a significantly higher content of anthocyanins primarily consisting of C3G, cyanidin-3,5-*O*-diglucoside, and cyanidin-3-*O*-rutinoside [7,13]. Haskap berries have a greater amount of anthocyanins (>200 mg/100 g FW) compared to other berries, such as cranberries, Saskatoon berries, or high-bush blueberries (<100 mg C3GE/100 g FW) [14]. 

A great body of research has focused on revealing the link between bioactive phytochemicals such as anthocyanins and human gut microbiota, mainly probiotics, in disease prevention. As has been revealed, bioactive phytochemicals play an extensive and critical role in shaping the gut microbiota to gain beneficial health effects by modulating gut microbiota-mediated cellular and molecular processes [15]. Human gut bacteria use C3G as substrates to form metabolites that can exert unique bioactive functions [16]. On the other hand, acting as prebiotics, anthocyanins modulate the growth of gut microbiota and improve the relative abundance of probiotics such as *Bifidobacterium* and *Akkermansia*, which are believed to be associated with anti-inflammatory effects [17]. As has been recently reported, the dietary supplementation of haskap berry pomace mitigated the dextran sulfate sodium (DSS)-induced colitis by improving the intestinal barrier integrity and modulating the gut microbiota [18]. Similar effects of haskap berries on restoring the balance of gut microbiota have been reported in murine obesity models as well [19,20]. 

However, due to the low bioavailability of anthocyanin and poor stability of probiotics in the gastrointestinal tract, the pronounced health benefits of these compounds are minimized. The challenge of bioavailability can be overcome by microencapsulation, a widely used technique in the food, pharmaceutical, and cosmetic industries. In this study, we used freeze-drying as the method of microencapsulation and maltodextrin and inulin as coating materials. The use of probiotic cocktails against colitis models is an emerging approach [21]. Hence, we utilized a mixture of common probiotic bacteria consisting of *Lactobacillus* and *Bifidobacterium* probiotic bacteria in the current study. We speculate that the combination of anthocyanins with probiotics holds promise to attenuate colitis in a more effective manner. Thus, this study is aimed at improving the bioavailability of haskap berry anthocyanins using microencapsulation and then assessing the anti-inflammatory potential of haskap berry anthocyanins with/without probiotics using the DSS-induced colitis of mice. 

## 2. Materials and Methods

### 2.1. Materials

Well-ripened haskap berry fruits (°Brix value of 16.8, variety Tundra) frozen at −20 °C were used in the process of anthocyanin extraction. Maltodextrin was purchased from ProteinCo Canada (Quebec City, QC, Canada), and inulin (chicory root fiber) was purchased from Sensus, Royal Cosun company (Lawrenceville, NJ, USA). Dextran sulfate sodium colitis grade (MFCD0008155, molecular weight 36,000–50,000) was purchased from MP Biomedicals (Irvine, CA, USA). The 11-strain probiotic powder was purchased from Custom Probiotics Inc. (Glendale, CA, USA). The primary antibodies used in Western blot analysis were purchased from Cell Signaling Technology (Danvers, MA, USA): anti-claudin-2 (E1H90, affinity purified rabbit polyclonal), mAb 48120, anti-BCL-2 (D17C4) rabbit mAb (mouse) 3498, anti-BAX (D3R2M) rabbit mAb (Rodent) 14796, anti-IL-6 (D5W4V) XP^®^ rabbit mAb (Mouse specific) 12912, anti-TNF (Cat: 3707), and anti-rabbit IgG, HRP-linked antibody (Cat: 7074). Anti-claudin-3 (ab15102), anti-claudin-4 (ab15104), anti-occludin (ab222691), and HRP-linked anti-*β*-actin antibody (mAbcam 8226) and loading control (ab20272) were purchased from Abcam Inc. (Toronto, ON, Canada). Alanine transaminase colorimetric activity assay (700260-96) and aspartate aminotransferase colorimetric activity assay kits (701640-96) were purchased from Cedarlane (Burlington, ON, Canada). 

### 2.2. Animals 

Ethical approval for animal use was obtained from the Dalhousie University Committee on Laboratory Animals (Protocol number: 19-098), which in turn applied the guidelines of the Canadian Council on Animal Care. Six-week-old male BALB/c mice were purchased from Charles River Canada (Lasalle, QC, Canada). Animals were housed in the Carleton Animal Care Facility, Tupper Building, Dalhousie University, Halifax, NS, Canada, and fed on a sterilized regular chow diet; water was supplied ad libitum. 

### 2.3. Preparation of Anthocyanin-Rich Haskap Berry Fraction 

Berries were freeze-dried at −40 °C for 72 h (Dura-Dry™ MP FD-14-85BMP1, (DJS Enterprises, Markham, ON, Canada); then, anthocyanin extracts were prepared according to a method described previously [22]. The ground berry powder was mixed with extraction solvent (80% ethanol, 0.5% formic acid, 19.5% distilled water) at a ratio of 1: 25 (*w*/*v*) and sonicated for 20 min at 35 ± 2 °C in an ultrasonic bath of 20 kHz/1000 Watts (model 750D, VWR, West Chester, PA, USA). The resulting ethanolic extract was filtered through P2-grade filter papers (09-805-5C, Fisher Scientific, Ottawa, ON, Canada) under vacuum and semi-dark conditions and concentrated (reduced the initial volume of 17 L to 2 L) using a rotary evaporator (Heidolph RotaChill, UVS400-115, Thermo Electron Corporation, Milford, MA, USA) maintaining a temperature of 45 °C in the water bath and −9 °C chiller temperatures at a speed of 80–100 rpm. The extracts were purified to obtain sugar-free anthocyanin extracts by solid-phase column chromatography. The column loaded with Sepabeads Resin (cat # 207-1, Sorbent Technologies, Atlanta, GA, USA) was preconditioned with deionized water and equilibrated overnight using 50% aqueous ethanol. About 1 L of concentrated anthocyanin extract was loaded into the column. The water-soluble sugar fraction was slowly eluted with deionized water. During the washing step, the Brix value of eluent was measured (≤0.1) using a refractometer (Digital refractometer 300016, SPER SCIENTIFIC, Scottsdale, AZ, USA) to confirm the removal of sugar. The bound anthocyanins were eluted using 95% ethanol after the sugar removal. Anthocyanin-rich haskap berry fraction (AHF) was prepared by rotary evaporation followed by freeze-drying of the elute. AHF was stored at −80 °C at dark in airtight containers until use.

### 2.4. Preparation of AHF Microparticles 

Maltodextrin (MD) and inulin (IN) were added at a ratio of 3:1 to 5 mL of deionized water, and the mixture was stirred for 90 s and kept overnight at 4 ± 2 °C. The wall material solution was then mixed with AHF powder (adhering to four different wall/core material ratios of 1:1, 1:1.5, 1:2, and 1:3) to obtain 40% of the final solid content. The resulting mixture was subsequently frozen at −20 °C and freeze-dried (−45 °C for 72 h) [23]. 

### 2.5. The Mixture of Probiotic Bacteria 

The mixture of probiotic bacteria used consists of 11 strains of Lactobacillus and Bifidobacterium comprising *Lactobacillus acidophilus* (LA-1), *L. rhamnosus* (LR-32), *L. salivarius* (LS-33), *L. plantarum* (LP-115), *L. casei* (LC-11), *L. lactis* (LL-23), *Bifidobacterium breve* (BB-03), *B. infantis* (Bi-26), *B. longum* (BL-05), *B. bifidum* (Bb-06), and *B. lactis* (BL-04). The 11-strain probiotic mixture, as a lyophilized powder, was provided by Custom Probiotics Inc., Glendale, CA, USA. 

### 2.6. Determination of the Encapsulating Parameters of Microparticles 

To determine the encapsulation efficiency, the total anthocyanin content (TAC) and surface anthocyanin content (SAC) were determined [24]. To obtain the TAC, 100 mg concentrations of microparticles were weighed, and 1 mL of deionized water was added. It was mixed well and sonicated at 35 °C for 1 min, and then 1 mL of formic acid and 9 mL of absolute ethanol were added followed by sonication for 5 min at 35 °C. The content was thoroughly mixed and centrifuged at 5000× *g* for 10 min. The supernatant was collected, and a 10% diluted sample was prepared by adding absolute ethanol. To determine the SAC, microparticles (100 mg) were added with 10 mL of absolute ethanol and vortex-mixed for 10 s, followed by centrifugation at 3000× *g* for 3 min. The supernatant was collected and used in TAC quantification. TAC and SAC were determined using the pH differential method using potassium chloride buffer at pH 1.0 (0.025 M) and sodium acetate buffer at pH 4.5 (0.4 M). The samples and buffers were mixed in a ratio of 1:4 and vortex-mixed for a few seconds, and the absorbance was measured at 520 and 700 nm [25]. The TAC was calculated as the C3G equivalents (C3GEs) according to the following equation:

TAC (mg C3GE/L) = ∆Aε × 1 × M × 10^3^ × D

where ∆A = (A520 pH 1.0–A700 pH 1.0) − (A520 pH 4.5–A700 pH4.5); ε (molar extinction coefficient) = 26,900 L/mol/cm for C3G; 1, path length in cm; M (molecular weight) = 448.8 g/mol for C3G; D, dilution factor; 10^3^, conversion from gram to milligram. The following equation was used to calculate the encapsulation efficiency using the results from the TAC and SAC contents:

$$Encapsulation\;efficiency\;\%=\frac{\left(TAC-SAC\right)}{TAC}*100$$

The encapsulation yield, anthocyanin recovery, and retention were calculated according to the following equations [26]:

$$Encapsulation\;yeild\;\%=\frac{Amount\;of\;anthocyanin\;entrapped\;in\;microparticles\;(mg)}{Total\;powder\;weight\;(mg)}*100$$

$$Anthocyanin\;recovery\;\%=\frac{Totala\;nthocyanin\;in\;microparticles\;(mg)}{Anthocyanin\;in\;the\;feed\;solution\;(mg)}*100$$

$$Anthocyanin\;retention\;\%=\frac{Actual\;loading}{Theoretical\;loading}*100$$

$$Actual\;loading=\frac{Mass\;of\;anthocynain\;in\;dried\;powder\;(mg)}{Total\;mass\;of\;the\;powder\;(mg)}$$

$$Theoretical\;loading=\frac{Mass\;of\;anthocynain\;in\;the\;feed\;solution\;(mg)}{Total\;mass\;of\;the\;feed\;(mg)}$$

### 2.7. Profiling and Quantification of Anthocyanin in Microparticles 

The powder products were analyzed using ultra-pressure liquid chromatography (UPLC)–electrospray ionization (ESI)–mass spectrometry (MS) (Waters, Milford, MA, USA) [27]. Briefly, powders were dissolved in ethanol containing 0.5% formic acid to obtain a final concentration of 1 mg/mL. It was mixed well and sonicated at 35 °C for 5 min and then centrifuged at 5000× *g* for 10 min. The supernatant was carefully collected and filtered through 0.22 μm nylon syringe filters. An Aquity BEH C_18_ (100 mm × 2.1 mm, 1.7 μm) column (Waters, Milford, MA, USA) was used. For the analysis of anthocyanin compounds, ESI in positive ion mode (ESI+), with a capillary voltage of 3000 V, nebulizer gas at 375 °C, and a flow rate of 0.35 mL/min was used. Anthocyanins were identified using the single-ion monitoring mode using specific *m*/*z* 449 for C3G, 661 for cyanidin-3,5-diglucoside, 463 for peonidin-3-glucoside, and 595 for cyanidin-3-*O*-rutinocide [28]. 

### 2.8. Determination of Physical Characteristics of microparticles 

#### 2.8.1. Moisture Content and Water Activity (aW) 

The moisture content of the microparticles was determined using a moisture analyzer (A&D MF-50, Wood Dale, IL, USA), and water activity was determined using a water activity meter (AQUALAB 4TE, Metergroup, Pullman, WA, USA). 

#### 2.8.2. Hygroscopicity 

The hygroscopicity of powder was measured under saturated NaCl solutions (Relative humidity of 75%) in desiccators. Samples were weighed after 1 week, and hygroscopicity was expressed as g of absorbed moisture per 100 g of dry solids [29]. 

#### 2.8.3. Water Solubility Index (WSI) 

The WSI of powders was measured by dissolving 0.5 g samples in 50 mL of distilled water and stirring at room temperature for 30 min. The suspension was then transferred to a 50 mL falcon tube and centrifuged at 3000× *g* for 5 min. Aliquots of the supernatant were then transferred to pre-weighed Petri dishes and dried at 105 °C for 5 h; then, final weights were measured. 

#### 2.8.4. The Bulk Density (ρbulk)

The ρbulk of the powders was measured by weighing 1 g of the sample and placing it in a 10 mL graduated cylinder. The cylinder was tapped by hand, and the bulk density was calculated as the ratio between the mass of powder contained in the cylinder and the volume occupied [30].

#### 2.8.5. Powder Morphology and Particle Size 

A scanning electron microscope (SEM) was used to study the outer structural appearance of the microparticles. The samples were attached to stubs using a two-sided adhesive tape and sputter coated with gold/palladium combination (4–10 nm) for 100 s at 30 mA, which was operated at 15 kV. The size of the microparticles was determined using the particle size analyzer (HoriBA, Partica LA-960 V2, Burlington, ON, Canada). 

### 2.9. Fourier-Transform Infrared (FTIR) Spectroscopy

FTIR analysis of microencapsulated particles and wall materials was performed using an FTIR spectrophotometer (Spectrum Two, PerkinElmer, Waltham, MA, USA). The wavelength range was set from 4000 to 400 cm^−1^, while the resolution was 4 cm^−1^.

### 2.10. In Vivo Experimental Design

The experiment was designed to determine the effect of five different dietary interventions on DSS-induced acute colitis in mice compared to regular chow with no DSS administration (control) and regular chow with DSS administration (disease model) groups. Considering the fact that 2 g of anthocyanin/human/day is the safe limit of anthocyanin for therapeutic effects, a dietary supplement for a 20 g BALB/c mouse was estimated to be 6.2 mg C3G equivalents/mouse/day, which is calculated according to the formula:

$$Human\;equivalent\;dose\;\left(\frac{mg}{kg}\right)=Animal\;dose\;(\frac{mg}{kg})\times \frac{Animal\;Km}{Human\;Km}$$

where the Km factor for mice and adult humans is 3 and 37, respectively [31]. The powder weight of the probiotic supplementation was determined considering the dose of live bacteria as 1 × 10^9^ CFU/g of probiotics/mouse/day. Mice were housed individually in filter-topped plastic cages with cellulose-based chips and paper strands and maintained under a 12 h light–dark cycle. After one week of acclimatization, mice were randomly divided into one of seven dietary groups (*n* = 5 in each). Respective diets were introduced on day 8 (e.g., after 7 days of acclimatization), and besides the control group, which received sterile drinking water, all other groups were supplied with 3% *w*/*v* DSS-added sterile drinking water from days 13th to 19th. Body weight, food intake, and water consumption were monitored throughout the experimental period. Starting from the 3% DSS administration, stool consistency was recorded and evaluated for occult blood using Hemoccult blood test strips (Hemoccult II, Beckman-Coulter, Brea, CA, USA). At the end of the treatments, mice were euthanized by isoflurane overdosing. 

#### 2.10.1. Organ and Blood Harvesting

Portal vein blood was collected using a 1 mL needle. Blood samples were incubated at room temperature for 1 h and then centrifuged at 14,000× *g* for 15 min at 4 °C, and the serum was stored at −80 °C until further analysis. The colon was isolated by cutting at the ileocecal junction and the distal end of the rectum, and the total length was measured. Colon contents were removed, weighed, and partitioned into two sections longitudinally. One section was processed into Swiss roll, and the other was snap-frozen and stored at −80 °C until further processing. The spleen was harvested, weighed, snap-frozen, and stored at −80 °C. The spleen index was calculated as spleen weight (mg) to body weight (g) ratio [32].

#### 2.10.2. Disease Activity Index (DAI)

The DAI was used to grade any morbidity due to inflammation. A score was derived from a compilation of relative body weight loss, stool consistency, and the presence of occult blood in the stool [33]. 

#### 2.10.3. Hepatotoxicity 

The levels of alanine aminotransferase (ALT) and aspartate aminotransferase (AST) in serum were measured using the ALT colorimetric activity assay kit (700260, Cayman CHEMICAL, Ann Arbor, MI, USA) and AST colorimetric activity assay kit (701640, Cayman CHEMICAL, Ann Arbor, MI, USA), respectively, by following the manufacturer’s instructions.

#### 2.10.4. Measurement of Protein Expression in Mice Colon Using Western Blot Analysis

To measure levels of protein levels of IL-6, TNF, bcl-2-like protein 4 (BAX), and B-cell lymphoma 2 (BCl-2), the total protein was extracted from mice colon tissues using radio-immunoprecipitation assay buffer including phosphatase and protease inhibitors and the concentration of the isolated protein quantified using Pierce™ Coomassie (Bradford) protein assay kit (Cat: 23200, Thermo Fisher Scientific, Rockford, IL, USA). Sample corrected to the same concentration were separated using 4–20% precast polyacrylamide gels (#4568095, Mini-PROTEAN^®^ TGX Stain-Free™ Protein Gels, Bio-Rad Laboratories Inc., Hercules, CA, USA) by electrophoresis and then transferred to polyvinylidene difluoride membranes. After blocking with 5% non-fat milk for 1 h, the membranes were incubated with antibodies to *β*-actin, BAX, BCL-2, TNF, IL-6, occludin, claudin-2, claudin-3, and claudin-4 (1:500) in 5% aqueous bovine serum albumin (BSA) solution at 4 °C for overnight while shaking. After the incubation, the membranes were washed with TBST buffer and probed with horseradish peroxidase (HRP)-linked anti-rabbit secondary antibodies (1:2000 *v*/*v* antibody dilution) in 5% aqueous BSA solution for 1 h at room temperature and then washed with TBST buffer and developed using enhanced chemiluminescence-based Clarity^TM^ and Clarity Max^TM^ Western ECL substrate kit (Cat: 1705060, Bio-Rad Laboratories Inc., Hercules, CA, USA). 

#### 2.10.5. Enzyme-Linked Immunosorbent Assays (ELISA)

Dilutions of homogenates of colonic tissues were prepared on ice and centrifuged, and the supernatants were assayed according to the ELISA kit instructions. Inflammatory markers (TNF, IL-1β, and IL-6) in mouse serum were measured using mouse TNF, SimpleStep ELISA^®^ kit (ab208348), mouse IL-1β, SimpleStep ELISA^®^ kit (ab197742), and IL-6 Mouse ELISA kit (ab46100) (Abcam Inc. Toronto, ON, Canada, respectively, according to the manufacturer’s instructions. All reagents were equilibrated to room temperature prior to use.

#### 2.10.6. Histological Analysis 

The Swiss roll preparations were immediately immersed into 10% acetate-buffered formalin for fixation for 24 h and then dehydrated in gradient alcohol in sequence, cleared in pure xylene, infiltrated with paraffin wax, and prepared into paraffin blocks. The wax block was cut to a thickness of 4 μm, hematoxylin–eosin (HE) staining was performed, and the tissue histology was evaluated by a pathologist (blinded to the treatment groups) following the colon histopathology scale (Table S1).

#### 2.10.7. Alpha Diversity of Fecal Microbiota

Effects of treatments on mouse gut bacteria diversity were investigated using the 16S rRNA sequencing technology at the Integrated Microbiome 15 January 2022). Raw data from DNA sequencing were analyzed by Qiime 2 (version 2020.8) microbiome bioinformatics software [34,35] and Shannon index, observed operational taxonomical units (OTUs), Faith’s phylogenetic diversity (PD), and Pielou’s evenness were calculated. 

#### 2.10.8. Statistical Analysis 

Graphical and statistical significance representations of data were generated using GraphPad Prism 9 (GraphPad Software, San Diego, CA, USA), and the values are represented as mean ± SD. Data were analyzed by one-way ANOVA followed by Tukey’s multiple mean comparison test or Bonferroni mean comparison and Pearson’s correlation test where applicable. *p* < 0.05 was considered to indicate a statistically significant result.

## 3. Results

### 3.1. Assessment of AHF Entrapment in MD and IN Matrix

**Figure 1:**
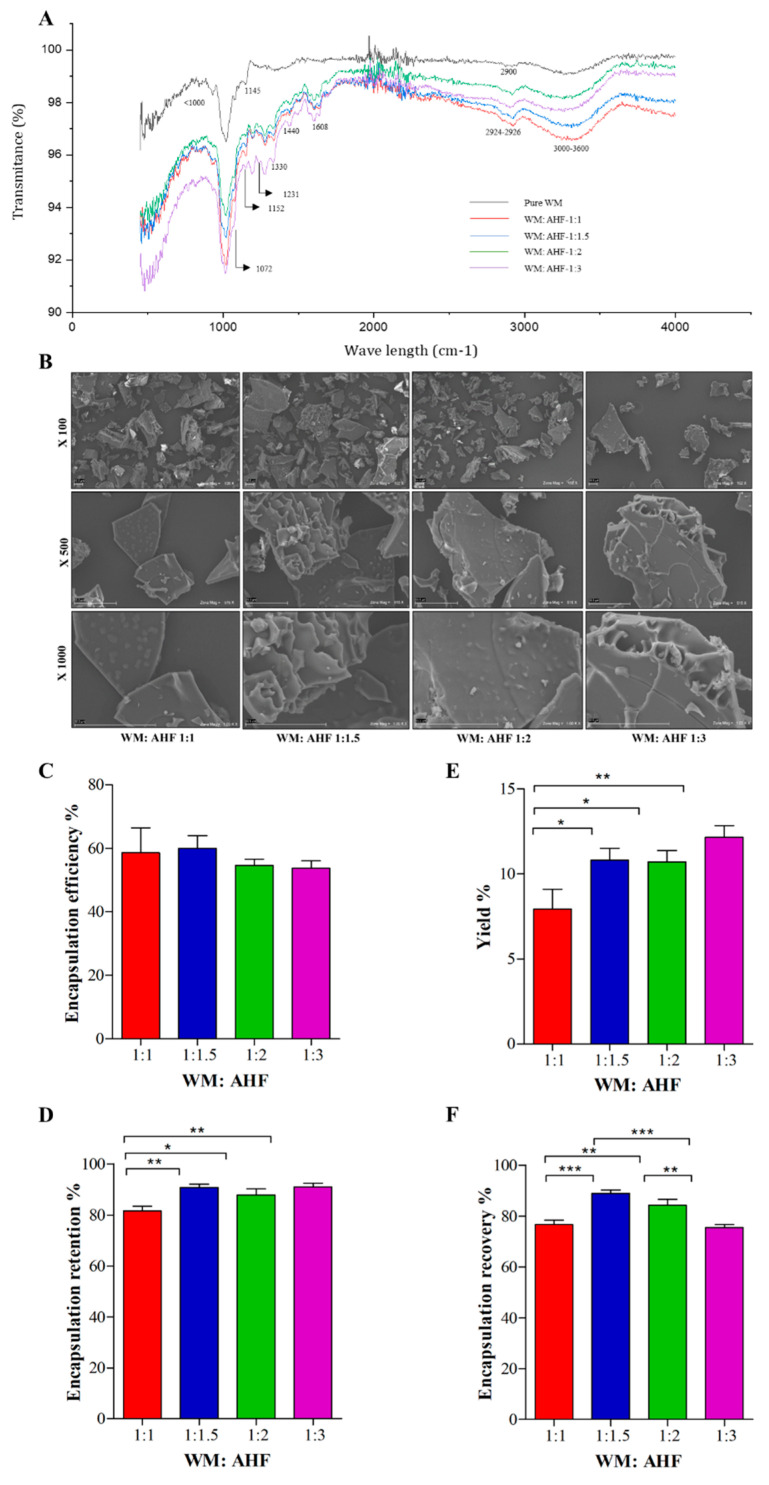
Physiochemical characteristics of microparticles prepared from anthocyanin-rich haskap fraction (AHF) using different amounts of wall materials: (**A**) FTIR spectra of microparticles. Wall material (WM) alone (black color spectrum), WM: AHF (1:1; red color spectrum, 1:1.5; blue color spectrum, 1:2; green color spectrum, and 1:3; purple color spectrum) are presented. Wall materials contained maltodextrin and inulin in a ratio of 3:1; (**B**) scanning electron microscopic images of AHF incorporated microparticles in three different magnifications (×100, ×500 and ×1000); (**C**) encapsulation efficiency %; (**D**) yield %; (**E**) encapsulation retention %; (**F**) Encapsulation recovery %. The means represent triplicates. For (**C**–**F**), a one-way analysis of variance was performed (*p* < 0.05) with Tukey’s multiple mean comparison test (at α = 0.05) for mean separation; *, **, *** indicate that differences among the compared groups were significant at *p* ≤ 0.05, *p* ≤ 0.01, and *p* ≤ 0.001, respectively.

The entrapment of anthocyanins in the MD-IN matrix was analyzed using FTIR spectra. Pure wall materials and wall materials together with AHF were tested separately (Figure 1A). Pure wall materials yielded characteristic peaks at <1000 cm^−1^, indicating the main infrared regions of MD, and near 1072 cm^−1^ and 1145 cm^−1^, indicating the C-O-C stretching ring vibrations of carbohydrates. In contrast, microparticles with AHF showed additional peaks around the regions of 1152 cm^−1^, 1231 cm^−1^, 1330 cm^−1^, 1440 cm^−1^, 1608 cm^−1^, and 2924–2926 cm^−1^ and broader and deep peaks around 3000–3600 cm^−1^ wavelengths. These results indicate that AHF was successfully loaded into the wall material matrix of MD and IN.

### 3.2. Anthocyanin Profiling and Physical Properties of Microparticles

The presence of anthocyanins in microparticles and freeze-dried AHF powder was determined using UPLC-ESI-MS (Table 1). Cyanidin-3-rutinoside, cyanidin-3-glucoside, cyanidin-3,5-diglucoside, and peonidin-3-glucoside were the four main anthocyanins detected. All four anthocyanins were significantly higher in the freeze-dried AHF powder (*p* < 0.05) than in microparticles. Among the anthocyanins, cyanidin-3-glucoside was observed in higher amounts than others in all tested ratios (*p* < 0.001). Physical parameters of microparticles were tested to select the best wall material (WM) to the core material ratio (WM:AHF ratios: 1:1, 1:1.5, 1:2, and 1:3) (Table 2). The moisture content of microparticles was between 6.9 ± 0.07 and 8.4 ± 0%. The WM:AHF ratio of 1:1.5 showed a significantly lower moisture % (*p* < 0.05), whereas the WM:AHF ratio of 1:3 showed the highest. By contrast, all tested ratios showed similar water activity (a_W_) in resulting microparticles. The least solubility was obtained for the microparticles produced using the 1:2 WM:AHF ratio, and the highest was observed with the 1:1.5 ratio (*p* < 0.05). Statistically similar solubilities were observed for the ratios 1:1 and 1:3. A significantly higher bulk density was detected for the microparticles of the 1:1 ratio (*p* < 0.05), and hygroscopicity was similar in powders of all four ratios. The particle diameter of the microparticles ranged between 37.5 and 2711 µm; however, their distribution was similar among the tested ratios. The external structure and morphology of AHF microparticles were observed under the SEM to visualize their shape. Microparticles produced with all four WM:AHF ratios resembled the broken glass structure of variable sizes, which is a common feature of freeze-dried powders. The particles showed irregular shapes, sharp edges, and deep grooves (Figure 1B). 

### 3.3. Assessment of the Encapsulation Productivity

The encapsulation productivity of microparticles was determined using four different parameters: encapsulation efficiency % (EE%), encapsulation yield % (EY%), encapsulation retention% (ERT%), and encapsulation recovery % (ER%) (Figure 1C–F). These parameters aid in determining the powder with minimum surface anthocyanins and the maximum capacity of the anthocyanins loading. In all four ratios, AHF encapsulating efficiency was above 50%. The encapsulation yield percentage (EY%) increased with the incorporation of AHF, reaching a peak at a WM:AHF ratio of 1:3 (*p* < 0.05). Notably, the WM:AHF ratio of 1:1 yielded the lowest encapsulation recovery percentage (ERT%) (*p* < 0.05), while other ratios resulted in similar ERT%. Moreover, ERT% tended to rise with an increase in AHF content in the encapsulation mixture. Regarding ERT%, particles from the 1:1 ratio exhibited lower values than those from the 1:1.5 and 1:2 ratios (*p* < 0.05). However, the highest ERT% was observed with microparticles having a WM:AHF ratio of 1:1.5. According to these findings, the WM:AHF ratio of 1:1.5 was selected as the most suitable candidate for AHF microparticles and tested in vivo.

### 3.4. The Clinical Response of Mice to Dietary Supplementations and DSS Administration 

**Figure 2:**
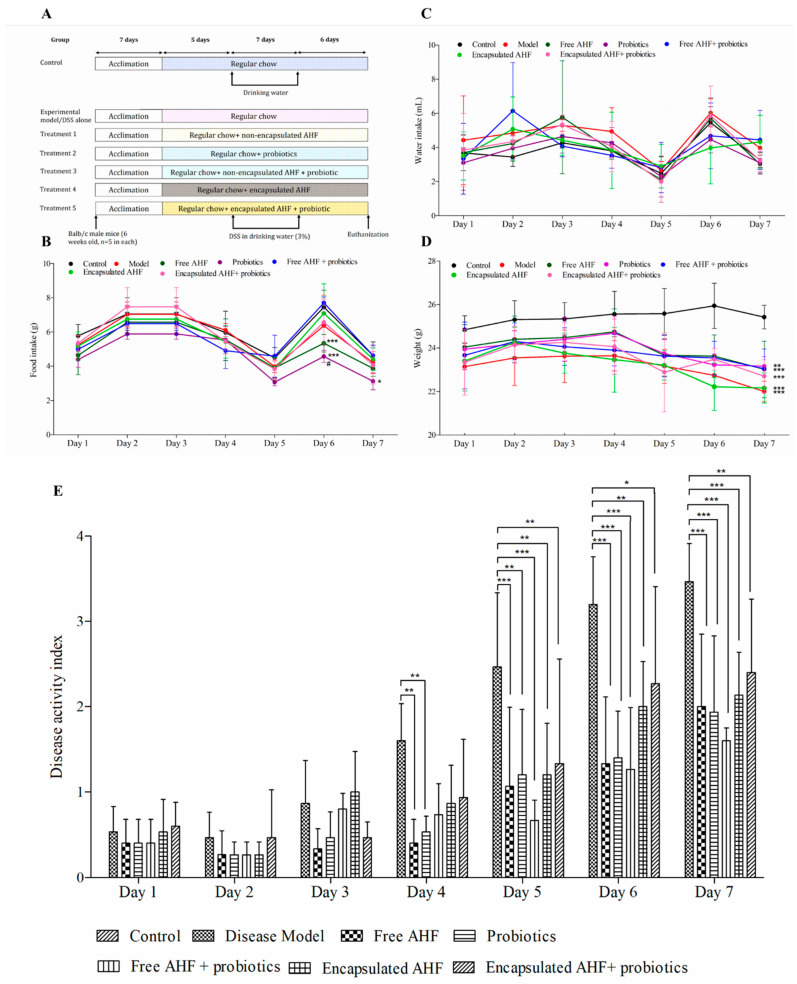
Effects of treatments on BALB/c mice receiving DSS: (**A**) schematic diagram of the animal experimental design with different dietary supplementary groups of male mice (*n* = 5); (**B**) food intake; (**C**) water intake; (**D**) body weight; (**E**) disease activity index (average of all mice within a treatment group score of: % weight loss, stool consistency, and presence of occult blood during DSS administration), all during the DSS administration period only. (**A**–**D**) Statistical analysis was performed using two-way ANOVA followed by Tukey’s multiple mean comparison test; * *p* ≤ 0.05, ** *p* ≤ 0.01, *** *p* ≤ 0.001 (compared to control), and # *p* < 0.05 (compared to the model) as indicated. (**E**) One-way analysis of variance was performed (*p* < 0.05) with Bonferroni pairwise comparison for mean separation; *, **, *** indicate that differences among the compared groups were significant at *p* ≤ 0.05, *p* ≤ 0.01, and *p* ≤ 0.001, respectively. DSS, dextran sulfate sodium; AHF, anthocyanin-rich haskap fraction.

The mice were divided into seven different dietary groups (Figure 2A). Food consumed during the DSS treatment followed the same fluctuating pattern in all treatment groups (Figure 2B). Food intake started to decline by day 3, and the least food intake was recorded on day 5 in all groups. Dietary supplementations with probiotics alone resulted in the lowest food intake during the recorded period in which it was significantly lower than the amount of the group free AHF + probiotic group at days 5, 6, and 7 (*p* < 0.05). In addition to that, on day 6, free AHF + probiotics showed higher food intake than the free AHF alone group (*p* < 0.05). Water consumption by each group during the exposure to DSS was measured to ensure that each mouse was exposed to the equivalent quantity of DSS. The water consumption of mice varied widely, yet any differences did not reach significance (Figure 2C). The body weight (g) of mice administered DSS and fed with regular chow (disease model) showed lower values than the healthy control during the assessment period (Figure 2D). The body weight of mice in the control group increased, while the body weight of the DSS group was reduced compared to the control group (*p* < 0.05). All dietary groups that received DSS showed a similar pattern in body weight reduction with their weight starting to decline on day 4, overlapping the trend in water consumption (which, interestingly, included the control group). The disease model group and other dietary groups that received DSS showed no significant difference in body weight (*p* > 0.05). However, there was a significant drop in the body weight of mice in dietary groups compared to the healthy control group on days 5, 6, and 7 (*p* < 0.05). The disease model group resulted in the lowest body weight on the final day of the DSS exposure period. The disease activity index (DAI), which was scored using % weight loss, stool consistency, and occult blood presence, is an indicator of the colitis severity that does not require euthanizing the animals. The disease model group had the highest DAI on all days, which was significantly higher than that of the healthy control group (*p* < 0.05) (Figure 2E). The DAI of the disease model group was higher than those of the groups of free AHF and probiotics on day 4 and all the treatment groups on days 5, 6, and 7 (*p* < 0.05). However, the lowest DAI score on all days, except on day 3, was obtained for the free AHF and probiotic supplementary group.

### 3.5. Dietary Supplementations and Macroscopic Measures of DSS-Induced Colitis

**Figure 3:**
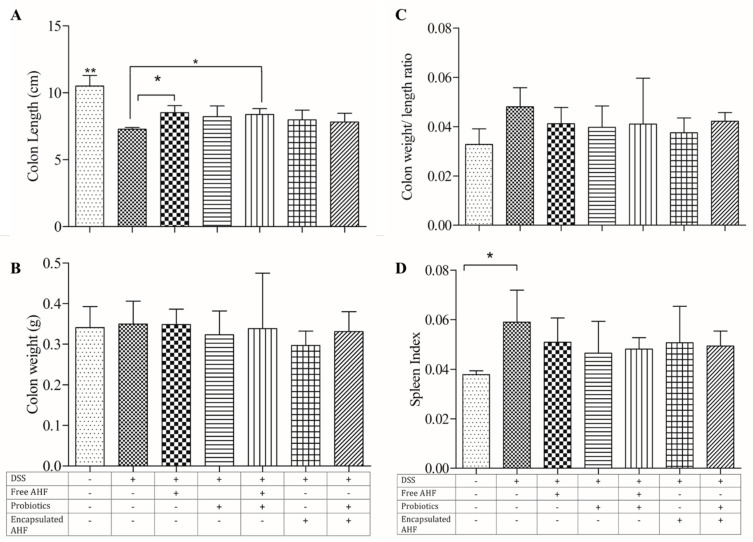
Gross measures of DSS-induced colitis in BALB/c mice on diets supplemented with anthocyanin-rich haskap fraction and probiotics, at necropsy: (**A**) colon length; (**B**) colon weight; (**C**) colon weight-to-length ratio; (**D**) spleen index. The treatments are indicated using + and – signs as presented in the table, in which + indicates the presence of the treatment and – indicates the absence of the treatment. One-way analysis of variance was performed (*p* < 0.05) with Bonferroni pairwise comparison for mean separation with *, ** indicating that differences among the compared groups were significant at *p* ≤ 0.05 and *p* ≤ 0.01, respectively. DSS, dextran sulfate sodium; AHF, anthocyanin-rich haskap fraction.

There was no mortality observed among any experimental group. Colons shortened with increasing inflammation in the DSS model. The mice in the healthy control group had the highest average colon length at 10.5 ± 0.79 cm (*p* < 0.05), while mice in the experimental model group had the lowest average length at 7.28 ± 0.13 cm, and the difference reached significance (Figure 3A). The average colon lengths of free AHF and free AHF + probiotic groups were significantly higher than the disease model group; however, these intestines remained significantly smaller than the healthy control (*p* < 0.05). There was no significant change in the colon weight and colon weight-to-length ratio of the mice among experimental groups (Figure 3B and Figure 3C, respectively). However, it was obvious that the colon weight-to-length ratio was increased in all DSS-treated groups compared to the control group. Compared to the control, the DSS-alone group showed a significant increase in spleen index (Figure 3D) (*p* < 0.05). The spleen index of other groups receiving DSS + dietary supplements was not different from the control group or the DSS-alone group. However, all the groups administered with DSS showed higher spleen index values than that of the control group, and the disease model had a significantly elevated spleen index than the control group (*p* < 0.05). 

### 3.6. Dietary Supplementations on Hepatotoxicity and DSS-Induced Colonic Inflammation

To assess whether the DSS administration or dietary supplementations cause any liver toxicity, serum ALT and AST amounts were examined (Figures S1A and S1B, respectively). ALT levels were recorded below the upper limit (0.06 U/mL) in all treatment groups. Regarding levels of AST in mouse serum, the disease model group showed a significantly elevated level compared to the healthy control, probiotics, and free AHF + probiotic groups (*p* < 0.05). Free AHF and free AHF + probiotic supplementations were more potent in reducing the serum AST levels in DSS-treated mice (*p* < 0.05). Comparatively, the combined effect of free AHF + probiotics in reducing the serum AST levels was significant (*p* < 0.05). Apart from the disease model and free AHF groups, other treatment groups showed no significant difference in serum AST levels compared to the healthy control (*p* > 0.05). DSS administration increases colonic apoptosis by upregulating BAX expression, downregulating BCL-2 protein expression, and elevating inflammatory cytokine expression. Colonic expressions of BCL-2, BAX, TNF-α, and IL-6 (Figure S2A–D) were analyzed by Western blotting, normalized to housekeeping protein β-actin, and presented as compared to the healthy control group. However, dietary supplementations were unable to reverse the effect of DSS significantly. Similarly, none of the diets significantly mitigated the effect of DSS on TJ protein expression (*p* > 0.05). 

### 3.7. Dietary Supplementations in Restoring the DSS-Induced Serum Inflammatory Cytokine Expressions

**Figure 4:**
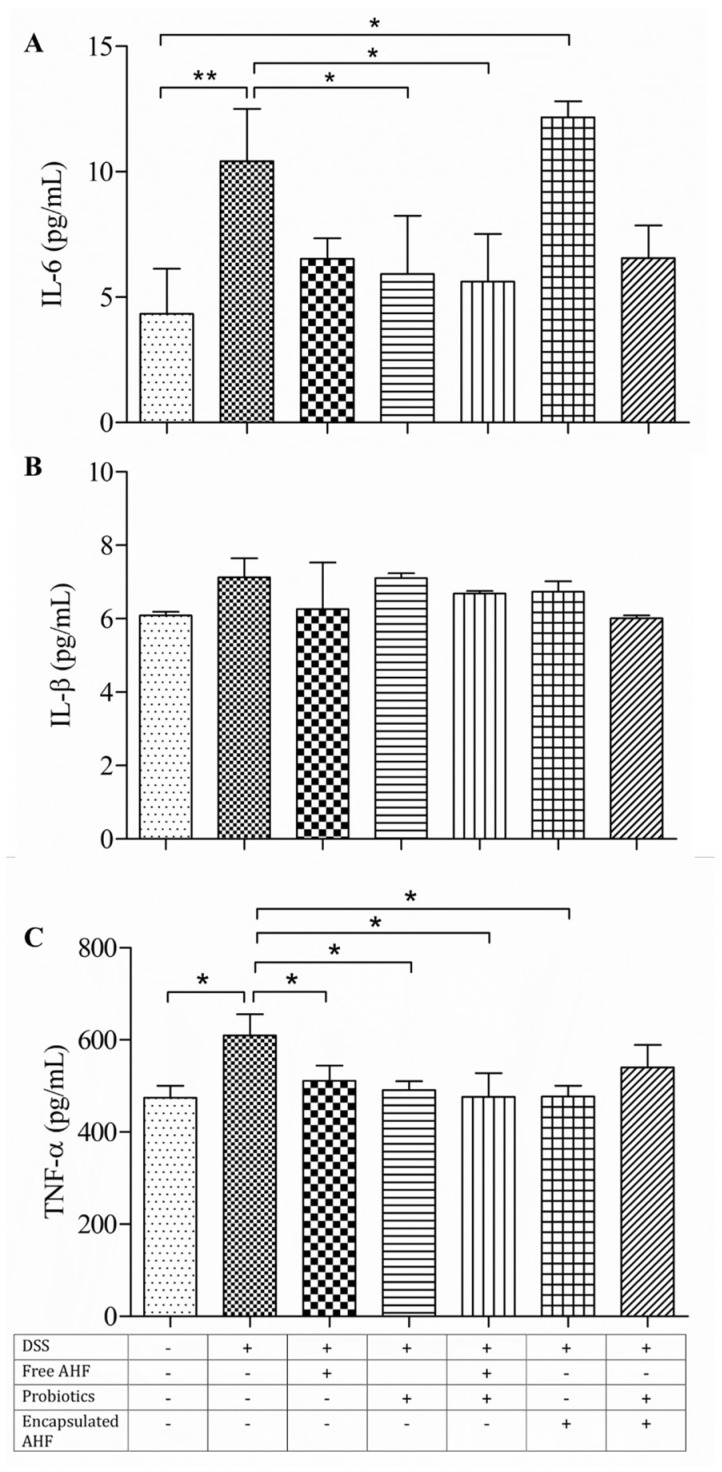
Effect of dietary supplementations on the levels of proinflammatory markers in BALB/c mice serum at necropsy: (**A**) IL-1β, (**B**) IL-6, and (**C**) TNF. The cytokines were measured by ELISA in triplicate, and the results are expressed as mean ± standard deviations, *n* = 5. The treatments are indicated using + and – signs as presented in the table, in which + indicates the presence of the treatment and – indicates the absence of the treatment. One-way analysis of variance was performed with Bonferroni pairwise comparison with *, ** indicating that differences among the compared groups were significant at *p* ≤ 0.05 and *p* ≤ 0.01, respectively.

In DSS-induced acute colitis, the mucosal epithelial barrier disruption enables luminal microorganisms to enter the mucosa, resulting in an inflammatory response including the overexpression of proinflammatory cytokines [36]. Here, serum inflammatory cytokine levels of IL-1β, IL-6, and TNF were determined (Figure 4A–C). The mean serum levels of IL-6 and TNF were higher in the disease model group compared to the healthy control and other dietary groups apart from the encapsulated AHF + probiotic group (*p* < 0.05). The serum IL-6 level of the encapsulated AHF group was significantly higher than the healthy control group (*p* < 0.05). However, the supplementary diets of probiotics and free AHF + probiotics reduced the serum IL-6 levels (*p* < 0.05), which were not different from the control group. Further, diets of free AHF, probiotics, free AHF + probiotics, and encapsulated AHF significantly reduced the DSS-induced elevation of serum TNF levels (*p* < 0.05). Neither DSS administration nor supplementary diets showed a change in serum IL-1β levels. 

### 3.8. Dietary Supplementation on Reducing DSS-Induced Colonic Inflammation 

**Figure 5:**
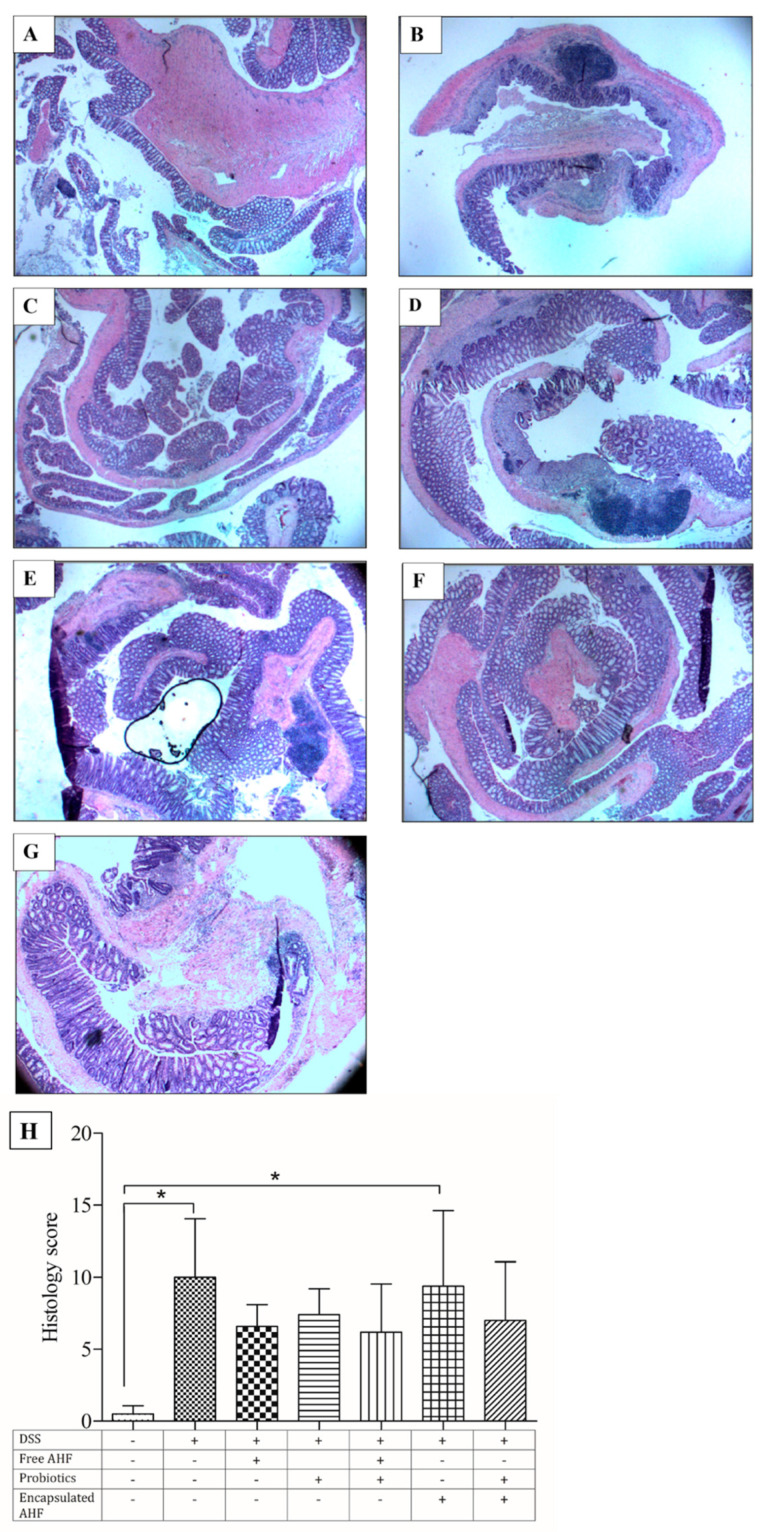
Histological examination of the pathology of colon Swiss rolls of DSS-treated BALB/c mice at necropsy: (**A**) control: regular chow, no DSS; (**B**) model: regular chow, DSS; (**C**) free AHF: regular chow with non-encapsulated AHF (6.2 mg C3GE/mouse/day), DSS; (**D**) probiotics: 11-strain probiotic powder (1 × 10^9^ CFU/mouse/day), DSS; (**E**) free AHF + probiotics: regular chow with non-encapsulated AHF (6.2 mg C3GE/mouse/day), 11-strain probiotic powder (1 × 10^9^ CFU/mouse/day), DSS; (**F**) encapsulated ACN: regular chow with encapsulated AHF (6.2 mg C3GE/mouse/day), DSS; (**G**) encapsulated anthocyanin + probiotics: regular chow with encapsulated AHF (6.2 mg C3GE/mouse/day), 11-strain probiotic powder (1 × 10^9^ CFU/mouse/day, DSS. Histology score (**H**) was calculated by combining the scores (maximum = 16) given for (a) leukocyte infiltration (0–5), (b) crypt damage (0–4), (c) ulceration (0–3), (d) edema (0, 1), and (e) crypt hyperplasia (0–3). The treatments are indicated using + and – signs as presented in the table, in which + indicates the presence of the treatment and – indicates the absence of the treatment. Kruskal-Wallis test was performed with Dunn’s multiple-comparison test; * indicates that differences among the compared groups were significant at α = 0.05. All images were obtained using 200× magnification.

**Figure 6:**
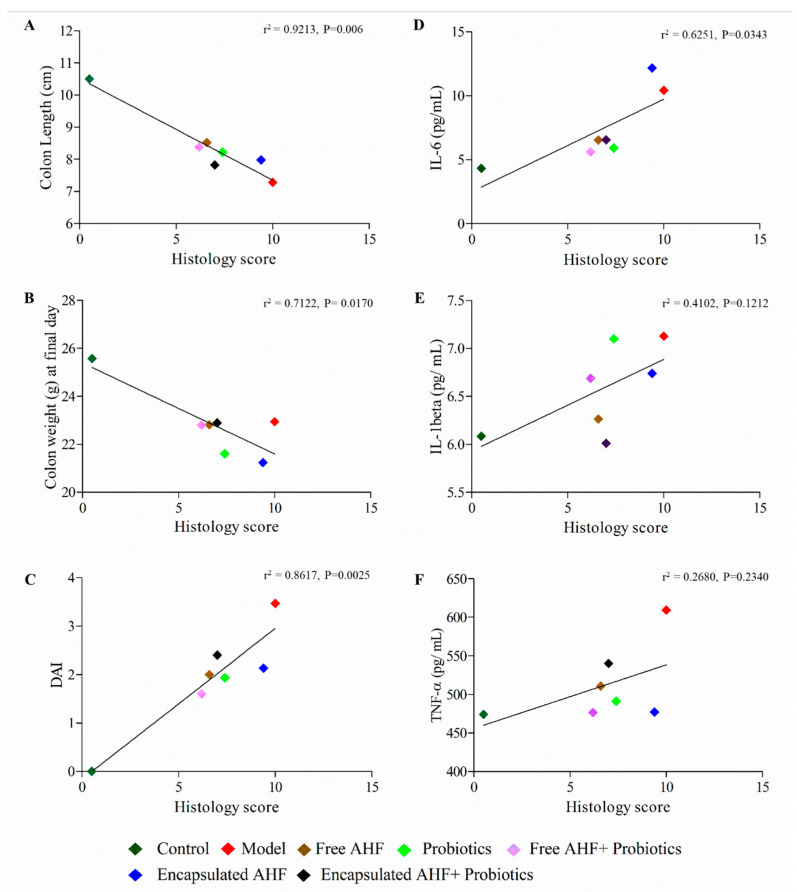
Linear regression analysis comparing histology score vs. (**A**) colon length, (**B**) colon weight at necropsy, (**C**) disease activity index (DAI) score, (**D**) IL-6 in serum, (**E**) IL-1 beta in serum, and (**F**) TNF in serum. DAI is described in the legend of Figure 5. r^2^, Pearson correlation coefficient.

H&E staining was performed to identify inflammatory changes in mice’s colon tissues. Histopathology scoring of the colonic tissues was carried out considering the edema, the presence of ulcers, crypt loss, neutrophil infiltration, and hyperplasia. Control mouse colon sections showed an intact epithelium, well-defined consistent crypt length, no edema or leukocyte infiltration in mucosa and submucosa, and no ulcers or erosions (Figure 5A). In contrast, colon tissue from DSS-treated mice showed increasingly severe inflammatory lesions extensively throughout the mucosa (Figure 5B–G). Compared to the healthy control group, the disease model group showed a significant increase in the pathology score (*p* < 0.05). All groups treated with DSS showed an increase in pathology scores compared to the healthy control group (Figure 5H). However, the group that received encapsulated anthocyanin registered a significant increase in the pathology score (*p* < 0.05). To better understand the relationship between histology and the other measures, linear regression analysis of histology score versus colon length, colon weight, DAI, IL-6, IL-1β, and TNF was performed (Figure 6). Predictably, colon length was inversely correlated with the pathology score with the Pearson correlation coefficient; r^2^ = 0.921 (*p* < 0.05). Likewise, colon weight was also inversely correlated with the pathology score (r^2^ = 0.712, *p* < 0.05). The DAI was positively correlated with the histology score (r^2^ = 0.862, *p* < 0.05). Similarly, serum IL-6 protein expression was also positively correlated with the histology score, producing an r^2^ of 0.625 (*p* < 0.05). However, neither serum IL-1β nor TNF levels were correlated with histology scores. 

### 3.9. Dietary Supplementation on DSS-Induced Disruption of Gut Bacteria

**Figure 7:**
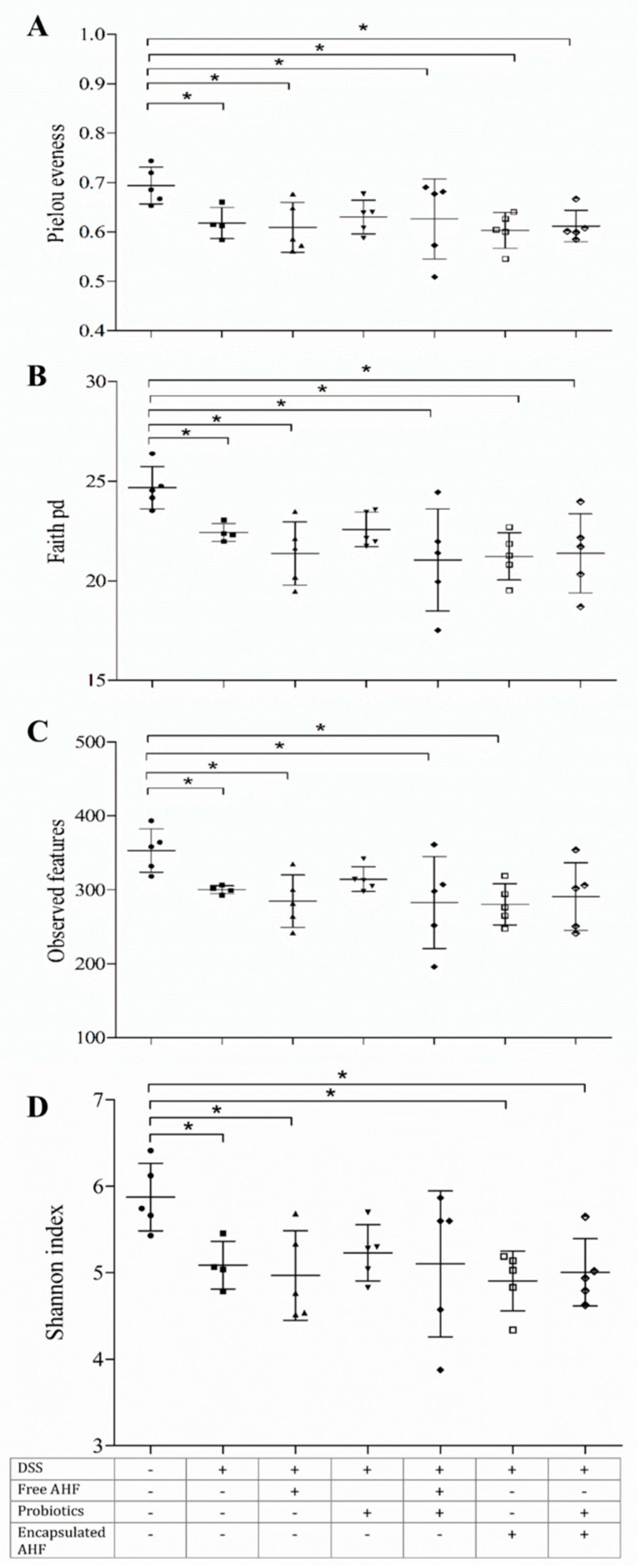
Effect of dietary supplementations on the fecal microbiome in BALB/c mice of DSS-induced colitis: (**A**) Pielou’s evenness, (**B**) Faith’s pd, (**C**) observed features, and (**D**) Shannon index. All parameters are displayed as a vertical scatter plot for each treatment group. The treatments are indicated using + and – signs as presented in the table, in which + indicates the presence of the treatment and – indicates the absence of the treatment. One-way analysis of variance was performed (*p* < 0.05) with Bonferroni’s multiple-comparison test (at α = 0.05) for mean separation; * indicates that differences among the compared groups were significant at α = 0.05.

The gut microbiota plays an essential role in maintaining the homeostasis of the host. As has been reported, the dysbiosis of the gut microbiome plays a crucial role in the pathogenesis of IBD [36]. The species richness and evenness of fecal microbiota of the DSS-induced colitis model were analyzed using 16S rRNA sequencing technology. The evenness of the microbiota was assessed in terms of Pielou’s evenness (Figure 7A). There was a significant reduction in microbial evenness in the DSS-alone group and dietary groups of free AHF, free AHF + probiotics, encapsulated AHF, and encapsulated AHF + probiotics compared to the healthy control group (*p* < 0.05). In contrast, mice fed with probiotics were found to have similar microbial evenness to that of the healthy control group. Faith phylogenic diversity was higher in the control group than in other treatment groups and was significantly different from all other groups (*p* < 0.05), except the probiotic group (Figure 7B). The administration of DSS significantly reduced the observed feature score in mice’s fecal microbiome (Figure 7C). However, diets of probiotics and encapsulated AHF + probiotics were able to restore the DSS-induced decline. When considering the alpha diversity in terms of Shannon index measures, the DSS-alone, free AHF, encapsulated AHF, and encapsulated AHF + probiotic groups showed a significant reduction compared to the control group (Figure 7D). However, the Shannon index values of the control and probiotic groups, as well as free AHF + probiotic groups, were not significantly different. Therefore, it was found that diets using probiotics and free AHF + probiotics are potent in restoring the gut microbiome disruption induced by DSS administration. 

## 4. Discussion

Anthocyanins, polyphenols found abundantly in colorful fruits and vegetables, have become an emerging food bioactive of interest in functional foods and nutraceuticals in recent decades due to their anticancer and anti-inflammatory properties [37]. However, the significantly low bioavailability of anthocyanins in the human body has led to the exploration of technologies such as microencapsulation to achieve their enhanced stability, bioavailability, and efficacy. The focus of the current study was to investigate the potential of microencapsulated or non-encapsulated AHF alone or in combination with probiotics in attenuating the severity of acute colitis. The majority of haskap anthocyanins consist of C3G [7]. C3G has been shown to have many physiological benefits in protecting gut health, including repairing the intestinal mucosal barrier and relieving inflammation by modulating the level of colitis-related indicators and signaling pathways [6]. In addition, C3G can be passed through the small intestine and biotransformed into metabolites such as PCA and PGA by colonic microbiota [38]. 

The microencapsulation of anthocyanins has the potential to improve the stability and bioavailability of anthocyanins. Instead of using a single WM, the use of WM combinations leads to higher encapsulation efficiencies due to multiple characteristics [39]. Maltodextrin (MD) has been used commonly in the microencapsulation of anthocyanins for their high holding capacity, low viscosity, and excellent solubility. However, to obtain microcapsules with improved mechanical stability and tailored microstructure, MD is commonly used in combination with other biomaterials [40]. We utilized a combination of MD and IN as WM in the microencapsulation of AHF. IN is a slightly branched fructooligosaccharide composed of β-(2→1)-linked fructose units, which are resistant to digestion in the human small intestine and pass to the large intestine and then broken down by bacteria, which can also generate short-chain fatty acids (SCFAs) with known mucosal protective effects [41]. 

The major anthocyanin found in AHF was C3G, and others were cyanidin-3-rutinoside, cyanidin-3,5-diglucoside, and peonidin-3-glucoside [28]. C3G content was significantly higher in the microparticles and AHF powder and accounted for 80% of the total anthocyanin content. The C3G content in haskap berries ranges from 75% to 90% [13]. When the amount of AHF was increased, the total anthocyanin content and C3G in the microparticles were increased, which might be due to the matrix differences of the WM used. 

The AHF-incorporated microparticles had desirable physical properties such as moisture content and a_W_. However, microencapsulated AHF has a broader range of particle sizes (D_10_ = 57.5 µm and D_90_ = 2711 µm). The highest particle size was observed with the least amount of AHF incorporated (WM: AHF, 1:1). As evident, the bulk density of the particles was proportional to the particle size. The particle diameter of spray-dried powders can range between 1 and 15 µm, whereas freeze-dried products can reach 300 μm [42]. 

The FTIR analysis of AHF microparticles predicts chemical bonding and interactions. Main infrared regions of MD below 1000 cm^−1^ correspond with C-O stretching vibrations of glucose rings. Vibrations around 1000 cm^−1^ are related to glucose monomers’ C-C pyranoid ring, C-O-C glycosidic bond, and C-OH side group in MD [43]. The 800–1200 cm^−1^ range represents carbohydrate fingerprint regions of fructofuranose ring vibrations in IN. The broadband at 3000–3600 cm^−1^ signifies O-H vibration groups in both MD and IN [44]. The WM and AHF spectra display bands at 1150 cm^−1^–1400 cm^−1^ for C-O stretching and C-O-H bending vibrations of phenols and flavonoids. The bands appearing between 1300 cm^−1^ and 1380 cm^−1^ indicate phenol deformations [45]. Increased intensities from 1500 cm^−1^ to 1700 cm^−1^ suggest the presence of anthocyanin. The changes in the 750–1000 cm^−1^ region indicate the presence of aromatic rings with ortho substitution [45]. An increase in the depth of the broader bands produced at 3000–3600 cm^−1^ is associated with the stretching vibration of O-H bonds in anthocyanins [46]. Freeze-dried microparticles exhibit broken glass structures without having a uniform surface, which are characteristics of microparticles produced by freeze-drying [47]. The heterogeneous surface of AHF microparticles was observed with multi-cavities and flake-like structures. A possible explanation for this phenomenon is the rapid sublimation of frozen water from the coating matrix, resulting in the formation of cavities where there were ice crystals before [48]. 

All AHF microparticles showed comparable high encapsulation efficiencies (54% to 60%), which suggests the compatibility of the MD and IN matrix in the encapsulation of AHF. Encapsulation efficiency was independent of the amount of AHF powder in the matrix mixture. However, encapsulating efficiency depends on the type and concentration of the WM [49]. Contradictorily, encapsulation efficiency can be dependent on the core/wall ratio as well [23]. The encapsulating yield and AHF retention were increased with an increase in the AHF amount in the encapsulating mixture. Concerning the anthocyanin recovery of the AHF microparticles, the results were independent of the AHF amount used in the encapsulating matrix. 

The DSS model of mice was used to investigate the effectiveness of AHF and probiotics in the prevention of acute colitis. DSS-induced colitis produces symptoms comparable to human gastrointestinal diseases such as body weight loss, diarrhea, bloody feces, and ulceration, more closely resembling ulcerative colitis. The appearance of clinical signs of DSS-induced colitis typically begins on days 3–4 of DSS exposure and gradually increases in severity over time [50]. The mice in the healthy or control group continued to increase their weight, whereas all DSS groups reported weight reductions during the DSS period. Free AHF and free AHF + probiotic treatments significantly inhibited the reduction in colon length in DSS-treated mice. The DSS causes an increase in spleen weight, which generally correlates with the severity of inflammation [51]. As per the current results, the supplementation of AHF and probiotics was able to reduce the spleen index values, mitigating the immunological alterations in mice spleen. 

The DAI is used to determine the severity of colitis. Consistent with previous studies, mice exposed to DSS developed body weight loss, watery diarrhea, and bloody stools [52]. The highest DAI values were observed with the disease model group. The supplementation of AHF and probiotics improved the DAI, which could be due to their effects on the inflammatory process, mucosal damage, and epithelial integrity or blockage of DSS molecules in accessing the colon epithelium. Colitis severity was mitigated by all dietary supplementations starting from day 5, providing evidence for inflammation preventive potential of AHF and probiotics by reducing the weight loss, increasing stool consistency, and modulating the barrier integrity. 

High doses of plant extracts, including anthocyanins, may confer health benefits but pose potential risks to liver and kidney function, with anthocyanin toxicity uncertain due to inadequate toxicological data for establishing safe daily intake levels [53]. On the other hand, DSS-induced inflammation can cause acute liver damage, which is reflected by elevated plasma levels of ALT and AST enzymes [54,55]. The serum levels of ALT were not significantly changed in the groups treated with DSS and supplemented with AHF (free and encapsulated forms) with/without probiotics from the control group, suggesting that 3% DSS or the amounts of anthocyanins and probiotics used in this study were not an effective influencer in acute liver injury. Similarly, some murine studies have shown that DSS alone does not influence serum ALT and AST levels; however, DSS with alcohol [56] and high-fat–high-carbohydrate diets [57] were hepatotoxic. In the present study, a significantly higher level of AST was found in the experimental model group and the supplementation of probiotics and AHF + probiotics significantly reduced the increase in serum AST levels. However, free AHF was not effective in reducing the AST levels. In contrast to our findings, anthocyanin extracted from black chokeberry showed a reduction in plasma ALT/AST levels in adult male Kunming mice in a dose-dependent manner after feeding for 8 weeks [58]. On the other hand, the administration of high doses of probiotics (1 × 10^10^ CFU) to healthy BALB/c mice treated with 2, 4, 6-trinitrobenzene sulfonic acid (TNBS) to induce acute colitis caused no toxicity [59]. Therefore, it can be confirmed that the amounts of anthocyanins (6.2 mg C3GE/mouse/day) and probiotics (1 × 10^9^ CFU) used in the present study were within safe limits for consumption. 

The imbalance between BCL-2 and BAX may cause cell apoptosis [60]. However, there are controversial findings on BCL-2 and BAX expressions in DSS-treated murine models, and there is no clear finding on how those protein expressions are changed. As we observed, DSS-induced colitis had no significant effect on BAX and BCL-2 changes, and that may be the reason why supplementary diets failed to improve those changes significantly. In contradiction to that, DSS-treated C57BL/6 mice fed with apple peel polyphenols [61], high hydrostatic pressure-treated C3G, and blueberry pectin complexes [62] significantly reduced the colonic tissue expression of BAX and improved the BCL-2 expression. 

There is growing evidence that the proinflammatory cytokines IL-6 and IL-1β play a crucial part in the uncontrolled colitis process [63,64]. A high level of IL-1β secretion by colon lamina propria monocytes was observed in patients with active IBD and high levels of IL-1β were associated with active lesions, which facilitated the localized inflammation [64]. In the present study, serum levels of TNF and IL-6 cytokines were significantly increased in the disease model group in comparison with the control group, with no detectable change in serum IL-1β and colonic expressions of TNF and IL-6 expressions. However, AHF and/or probiotic diets were potent in downregulating the serum TNF-α and IL-6 cytokine expressions. Similarly, diets of anthocyanin-rich powders such as Goji berry [65], Maqui berry [66] Aronia berry [67], and probiotics such as *Lactobacillus acidophilus* [68] are capable of reversing DSS-induced cytokine upregulation. Additionally, the gut microbiota impacts the immune response of the host, potentially causing an imbalance in cytokine levels. Specifically, TNF production capacity seems to be notably influenced by the microbiome, while other cytokines like IL-1β and IL-6 show fewer associations, albeit more specific, with the gut microbiota [69]. 

The colon epithelium maintains its selective permeabilities by limiting the passage of pathogenic bacteria and/or their toxic metabolites, chemical toxins, and antigens, as well as by permitting the absorption of nutrients, electrolytes, and water basically with the help of TJ proteins [70]. The DSS-induced changes in TJ expression are varied upon the dose of DSS and even in the same mouse strain [65,71,72]. To date, the exact mechanism by which DSS causes colitis and loss in TJ proteins is poorly identified. Considering the functionality of claudin-2, it is recognized as a pore-forming protein, whereas claudin-3 and claudin-4 are involved in the pore-sealing process [73]. In this context, the overexpression of claudin-2 should result in increasing intercellular permeability and leakiness, while the overexpression of claudin-3 and 4 enhances the paracellular barrier and reduces intercellular permeability. The dysfunction of TJ proteins, therefore, can cause colitis by weakening the intercellular adhesion and promoting intestinal permeability for pathogenic bacteria [74]. As observed in the current study, DSS or supplementary diets failed to induce significant differences in occludin and claudin-2, -3, and -4 expressions. Once the DSS treatment is stopped, colons can be regenerated as inflammation resolution/epithelial repair occurs [75]. 

Results of the H&E staining of colon tissue revealed that the epithelial cells of the control group were normal, as expected. The crypt structure was regular and with no spaces, damage, or hyperplasia. Further, a control group of mice colon tissues was found with no ulcers/inflammatory lesions, submucosal edema, or leukocyte infiltration. However, the colonic tissues of mice in the disease model group, which significantly differed from the control group, showed the typical architecture of severe inflammation, which was indicated by massive loss of crypts, crypt hyperplasia, large inflammatory lesions/ulcers in the submucosa, lack of surface epithelium, obvious leukocyte infiltration, and submucosal edema. Similar tissue architectures were reported for DSS-induced colonic destruction [65,66,71]. Moreover, the histology score of the present study was negatively correlated with colon length and colon weight, indicating that mice fed with free AHF + probiotics and free AHF tended to have higher colon length and lower histology score or lower disease severity with respect to the experimental model. On the other hand, the same feeding groups exhibited the least DAI and histology scores for other dietary groups and the experimental model group. As indicated, colonic cytokine levels (IL-6, TNF-α, and IL-1β) were increased with histology score providing a moderately positive relationship. 

The mice’s fecal microbial diversity was analyzed using 16S rRNA sequencing technology in terms of alpha diversity changes, attributed to four major ecological parameters, including Pielou’s evenness (to show how evenly the individuals in the community are distributed over different operational taxonomic units), Faith’s phylogenetic diversity (pd, the abundance of microbial population and includes the abundance of rare species), the observed features (richness of the microbial community), and Shannon index (the combined parameter of richness and evenness). The results of the present study showed that the treatment with 3% DSS resulted in a decrease in the species’ alpha diversity. However, Faith’s pd, observed features, and Shannon index values of mice fed with a probiotic diet appeared similar to that of the control group, whereas the groups with free AHF + probiotics and probiotics resembled Pielou’s evenness in the control group. 

The anti-inflammatory properties of haskap anthocyanins are mainly attributed to the metabolites generated in the human body. More than 20 C3G metabolites have been identified; however, PCA, vanillic acid, and ferulic acid and their derivatives are considered potential bioactive metabolites against inflammation. Under the regulation of gut microbiota, PCA and PGA further metabolized into microbial metabolites such as vanillic acid and ferulic acid [76]. The PCA has been reported to inhibit the production of inflammatory mediators, such as IL-6, TNF, IL-1β, and prostaglandin E2 (PGE2), potentially by suppressing the activation of NF-κB and extracellular signal-regulated kinase (ERK) in murine BV2 microglia cells and colitis mouse model. Vanillic acid can also inhibit the production of proinflammatory cytokines such as TNF, IL-6, IL-1β, and IL-33 by downregulating caspase-1 and NF-κB pathways [8]. The anti-inflammatory effects of probiotics have also been explored. The exact mechanism of how probiotics exert anti-inflammation is yet to be understood. However, as reported in many studies, probiotics are potent in reducing inflammation by producing the immunoregulatory factors that directly suppress proinflammatory cytokines by promoting the proliferation of regulatory T lymphocytes, resulting in cell contact-dependent immunoregulation and the secretion of anti-inflammatory cytokines, as well as by modulating gut microbiota [16]. 

As per the results obtained in the present study, encapsulated AHF was not as efficient as free AHF in the prevention of DSS-induced colitis, and this might be attributed to the use of IN. The use of IN in the prevention of DSS-induced colitis has controversial findings. As an example, seven days of pretreatment with IN also failed to improve DSS (3%)-induced colitis in C57Bl/6 mice [77]. However, the exact mechanism by which IN elevates DSS-induced inflammation is unknown. Contrarily, there are supportive studies on the anti-inflammatory effects of IN as well. In the current findings, the IN-induced elevation of colitis was not observed. Therefore, the role of IN in DSS-induced colitis is yet to be explored. Further, toxicology studies should be considered to investigate the IN-toxicity in the encapsulation of bioactive phytochemicals. However, free AHF and free AHF + probiotic diets mitigated the severity of DSS-induced acute colitis in BALB/c mice and could be a potential candidate for attenuating the CRC after conducting pharmacokinetics studies.

## 5. Conclusions

AHF microparticles were successfully encapsulated in the MD/IN matrix at a ratio of 1:1.5 using freeze-drying, resulting in a glass-like brittle structure. The mice fed on free AHF and free AHF + probiotics significantly reduced the DSS-induced colitis, as seen using multiple measures. For example, DSS-induced proinflammatory biomarkers, i.e., IL-6 and TNF, were reduced significantly by dietary supplementation of free AHF and free AHF + probiotics. The supplementary diets of AHF and AHF + probiotics showed a closer resemblance to alpha diversity in control mice. AHF microparticles were not effective in attenuating DSS-induced colonic inflammation. Further studies are needed to investigate alternative encapsulation methods of haskap anthocyanin to improve its efficacy in mitigating inflammatory response.

## Figures and Tables

**Table 1:** Anthocyanin content (mg/g DW) of AHF microparticles prepared from the different ratios of wall materials in comparison to AHF powder.

Anthocyanin	AHF-Incorporated Microparticles				AHF Powder
	WM: AHF 1:1	WM: AHF 1:1.5	WM: AHF 1:2	WM: AHF 1:3	
Cyanidine-3-rutinoside	1.2 ± 0.06 ^a^	1.3 ± 0.02 ^a^	2.5 ± 0.16 ^a^	16.7 ± 0.22 ^c^	19.2 ± 1.08 ^c^
Cyanidine-3-glucoside	17.0 ± 1.23 ^a^*	18.9 ± 0.59 ^a^*	38.7 ± 0.84 ^b^*	277.9 ± 10.86 ^c^*	281.5 ± 16.82 ^c^*
Cyanidine-3,5-diglucoside	1.2 ± 0.09 ^a^	1.6 ± 0.05 ^a^	3.3 ± 0.05 ^a^	23.7 ± 1.36 ^c^	27.2 ± 1.45 ^c^
Peonidine-3-glucoside	0.8 ± 0.04 ^a^	0.7 ± 0.05 ^a^	2.5 ± 0.04 ^a^	29.4 ± 0.43 ^c^	26.2 ± 1.90 ^c^

Wall materials (WM) of the microparticles consisted of maltodextrin and inulin in a 3:1 ratio. Anthocyanin-rich haskap fraction (AHF) microparticles were produced using four different WM:AHF ratios as 1:1, 1:1.5, 1:2, and 1:3. HPLC/MS analysis of different types of anthocyanins per gram of AHF microparticles and freeze-dried AHF powder was carried out. The results represent triplicates. One-way analysis of variance was performed (*p* < 0.001) with Tukey’s multiple mean comparison test (at α = 0.001) and Bonferroni pairwise comparison (at α = 0.05) for mean separation. Results that share the same superscript letter within the rows are not significantly different; * indicates the significant differences among the compared groups in the same column.

**Table 2:** Physical properties of microparticles prepared using anthocyanin-rich haskap fraction (AHF).

Wall Material: AHF Ratio	Moisture%	aW	Solubility%	Bulk Density (g/cm^−3^)	Hygroscopicity (g/100 g)	Particle Size (µm)		
						D_10_	D_50_	D_90_
1:1	7.3 ± 0.07 ^b^	0.6 ± 0.00 ^a^	95.8 ± 08 ^b^	0.3 ± 0.01 ^b^	21.7267 ± 0.94 ^a^	57.5	281	2711
1:1.5	6.9 ± 0.07 ^c^	0.6 ± 0.00 ^a^	97.9 ± 0.61 ^a^	0.2 ± 0.01 ^a^	21.2031 ± 0.22 ^a^	47.8	188	830
1:2	7.5 ± 0.07 ^b^	0.6 ± 0.00 ^a^	93.9 ± 0.09 ^c^	0.2 ± 0.01 ^a^	21.4202 ± 0.05 ^a^	37.5	138	445
1:3	8.4 ± 0.00 ^a^	0.6 ± 0.00 ^a^	95.6 ± 0.13 ^b^	0.2 ± 0.01 ^a^	21.7623 ± 0.20 ^a^	53.5	186	965

Wall materials (WM) of the microparticles consisted of maltodextrin and inulin in a 3:1 ratio. Anthocyanin-rich haskap fraction (AHF) microparticles were produced using four different WM:AHF ratios as 1:1, 1:1.5, 1:2, and 1:3. The results represent triplicates. One-way analysis of variance was performed (*p* < 0.05) with Bonferroni pairwise comparison (at α = 0.05) for mean separation. Results that share the same superscript letter within the columns are not significantly different. Abbreviations: D_10_ = particle diameter corresponding to 10% of the cumulative distribution; D_50_ = particle diameter corresponding to 50% of the cumulative distribution; D_90_ = particle diameter corresponding to 90% of the cumulative distribution.

## Data Availability

The original contributions presented in the study are included in the article/Supplementary Material, further inquiries can be directed to the corresponding author.

## Supplementary Materials

The following supporting information can be downloaded at: https://www.mdpi.com/article/10.3390/foods13131987/s1, Table S1: Colon histopathology scale (Swiss roll sections); Figure S1: Assessment of liver toxicity of Balb/c mice in 3% DSS-induced acute colitis model under several dietary interventions; Figure S2: Expression of (A) BCl-2, (B) BAX, and inflammatory cytokines (C) TNF-α and (D) IL-6 in Balb/c mice colon tissues; Figure S3: Effect of dietary supplementations on the expression of tight junction proteins in Balb/c mouse colon tissues in 3% DSS induced acute colitis model. (A) Occludin, (B) Claudin-2, (C) Caudin-3, and (D) Claudin-4. One-way analysis of variance was performed with Bonferroni pairwise comparison with *, ** indicating that differences among the compared groups were significant at *p* ≤ 0.05 and *p* ≤ 0.01, respectively.
