# Supplementary material for: Dietary Supplementation of Haskap Berry (Lonicera caerulea L.) Anthocyanins and Probiotics Attenuate Dextran Sulfate Sodium-Induced Colitis: Evidence from an Experimental Animal Model
Source: Foods. 2024 Jun 24;13(13):1987. doi: 10.3390/foods13131987 (PMC11241346; doi:10.3390/foods13131987)
Supplement: Supplementary file 1 [file foods-13-01987-s001.zip › foods-3022507-supplementary.pdf]

Table S1: Colon histopathology scale[1] (Swiss roll sections)

Score	Leukocyte infiltrate	Crypt damage	Ulceration	Edema	Crypt hyperplasia
**0**	**none**<br>(including lymphoid aggregates often seen in healthy colon)	**none**<br>(may be rare patch "empty" or vacuoles in healthy)	**none**<br>(intact epithelium, wary of histological artefacts)	**none**<br>(mucularis mucosa tight to mucos and muscle	**none**<br>(crypt heights generally uniform but may differ at different areas)
**1**	occasional patchy cells (limited to base of crypt lamina propria in IL-10ko, ± submucosal in DSS)	patchy crypt loss, spaces appear between crypts	small, focal ulcers (<3 total and <15 crypt widths)	present and obvious, (may be patchy, typically with cells)	1 or 2 patches visible at low power, < 75% higher than the average flanking crypts & boundary clear
**2**	significant presence of cells in lamina propria, limited to focal areas	1 or 2 long stretches lacking crypts, loss of goblet cells, some shortening of crypts	frequent small ulcers (>3 < 5 crypt widths, or 1 or 2 > 15 crypt widths)		1 or 2 patches ~75% higher than flanking crypts
**3**	infiltrate present in submucosa in focal areas	single long stretch, < 15 crypt widths	multiple large stretches lacking surface epithelium, not necessarily >50% length		>2 patches or ≥75% higher than flanking crypts, striking at low power
**4**	large numbers in submucosa, lamina propria and surrounding blood vessels, covering ~50% of colon length or involves proximal colon	multiple large stretches lacking crypts though < 50% colon length, typically obscured by infiltration or involves proximal (colon is "mushy")			
**5**	occupying ≥50% of colon length and/or transmural inflammation or abundant crypt abscesses (colon is mushy)				

[1] Updated January 31, 2019

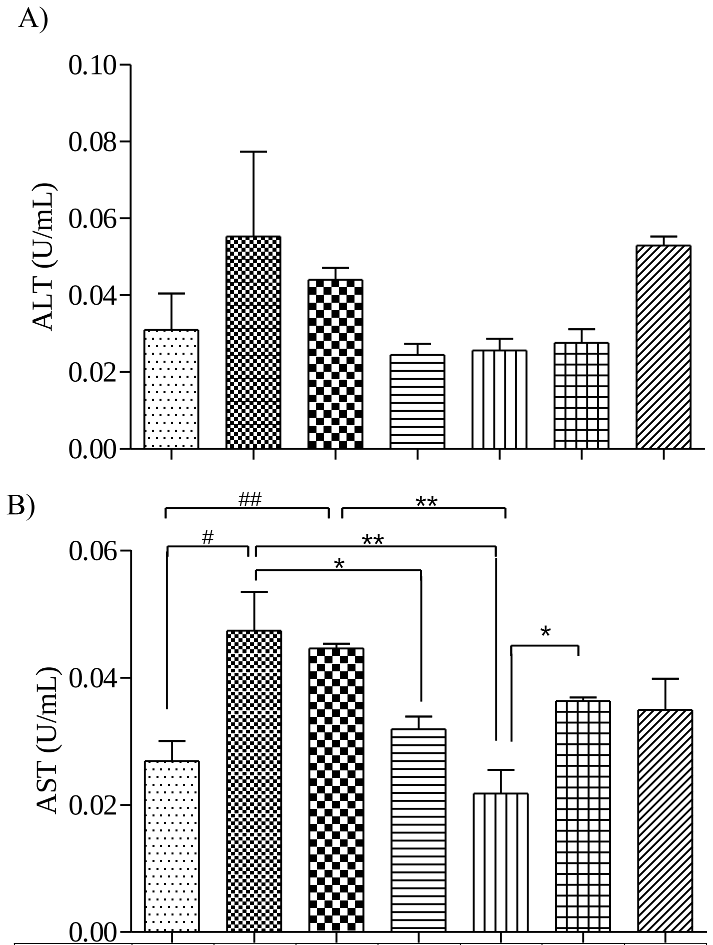

DSS	-	+	+	+	+	+	+
Free AHF	-	-	+		+	-	-
Probiotics	-	-	-	+	+	-	+
Encapsulated AHF	-	-	-	-	-	+	+

Figure S1: Assessment of liver toxicity of 3% DSS-induced BALB/c mice of acute colitis under dietary interventions.

Liver toxicity was expressed in terms of (A) alanine aminotransferase (ALT) and (B) aspartate aminotransferase (AST) in mice serum (U/mL). Dietary supplementary groups included control: regular chow, no DSS; model: regular chow, DSS; Free AHF: regular chow with non-encapsulated anthocyanin rich haskap fraction (6.2 mg/mouse/day), DSS; Probiotics: 11 strain probiotics powder (1×10^9 CFU/mouse/day), DSS; Free AHF + probiotics: regular chow with non-encapsulated anthocyanin rich haskap fraction (6.2 mg/mouse/day), 11 strain probiotics powder (1×10^9 CFU/mouse/day), DSS; Encapsulated AHF: regular chow with encapsulated AHF (6.2 mg/mouse/day), DSS; Encapsulated AHF + probiotics: regular chow with encapsulated AHF (6.2 mg/mouse/day), 11 strain probiotics powder (1×10^9 CFU/mouse/day, DSS, at which n=5 in each. One Way Analysis of Variance was performed ($p < 0.05$) with Tukey's multiple mean comparisons (at $\alpha = 0.05$) for mean separation. * Indicates that differences among the compared groups were significant at $\alpha=0.05$. Abbreviations: DSS, dextran sulfate sodium, AHF, anthocyanin-rich haskap fraction.

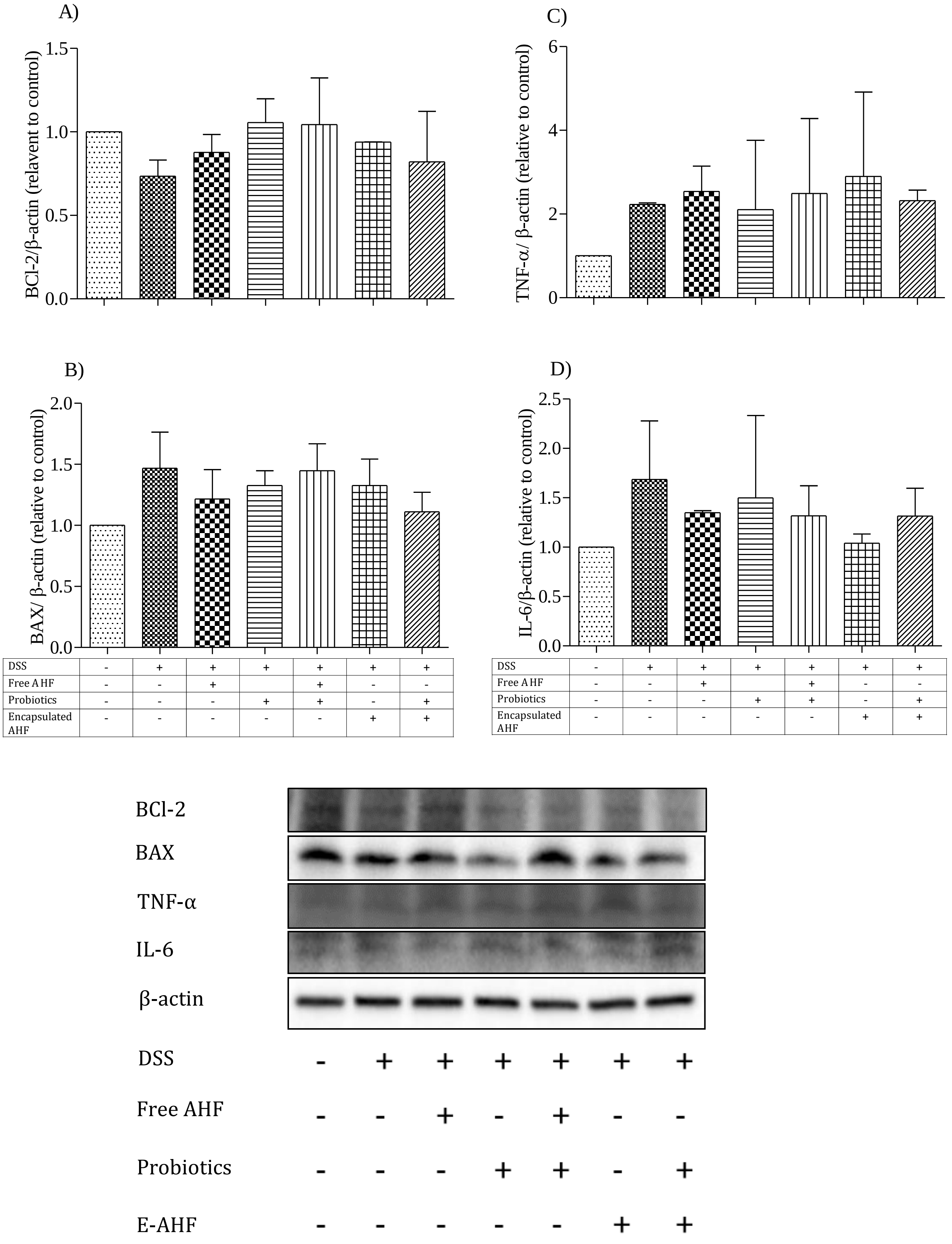
A)
BCl-2/β-actin (relavent to control)
1.5
1.0
0.5
0.0
C)
TNF-α/ β-actin (relative to control)
6
4
2
0
B)
BAX/ β-actin (relative to control)
2.0
1.5
1.0
0.5
0.0
D)
IL-6/β-actin (relative to control)
2.5
2.0
1.5
1.0
0.5
0.0
DSS - + + + + + +
Free A HF - - + + - -
Probiotics - - - + + - +
Encapsulated AHF - - - - - + +
BCl-2
BAX
TNF-α
IL-6
β-actin
DSS - + + + + + +
Free AHF - - + - + - -
Probiotics - - - + + - +
E-AHF - - - - - + +

Figure S2: Expression of (A) BCl-2, (B) BAX, (C) TNF-α and (D) IL-6 in BALB/c mice colon tissues.

Protein expression was determined using western blot analysis and images were analyzed using Image lab version 6.0.1 software. Results were normalized using the β-actin level and finally expressed as the relative protein levels compared to the control. At least three western blotting experiments were performed, and the results were expressed with means ± standard deviations. One Way Analysis of Variance was performed ($p < 0.05$) with Tukey's multiple mean comparisons ($\alpha = 0.05$) for mean separation. Abbreviations: Free AHF, non-encapsulated anthocyanin-rich haskap fraction, E-AHF, encapsulated anthocyanin, BCL-2, B cell lymphoma 2, BAX, Bcl-2-associated X protein.

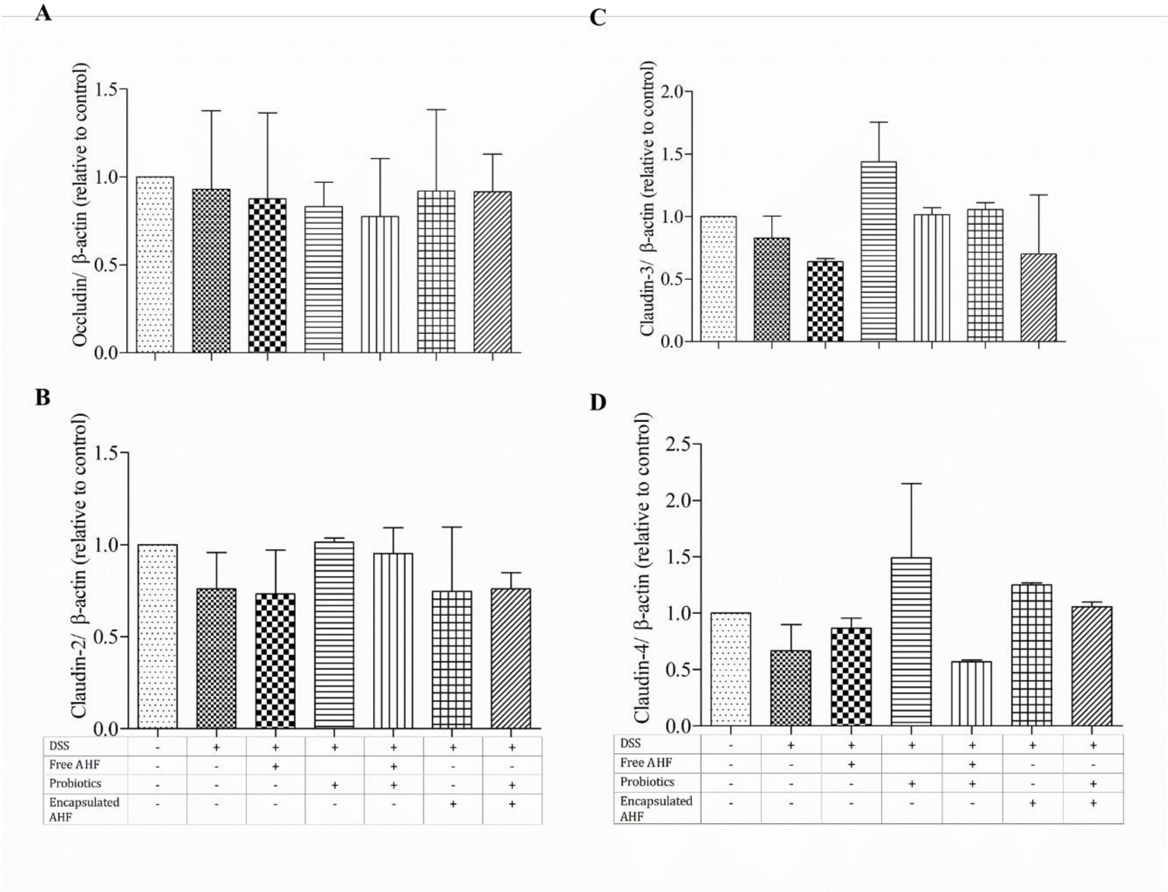
A
Occludin/ β-actin (relative to control)
0.0
0.5
1.0
1.5
C
Claudin-3/ β-actin (relative to control)
0.0
0.5
1.0
1.5
2.0
B
Claudin-2/ β-actin (relative to control)
0.0
0.5
1.0
1.5
DSS - + + + + + +
Free AHF - - + + - -
Probiotics - - - + + - +
Encapsulated AHF - - - - - + +
D
Claudin-4/ β-actin (relative to control)
0.0
0.5
1.0
1.5
2.0
2.5
DSS - + + + + + +
Free AHF - - + + - -
Probiotics - - - + + - +
Encapsulated AHF - - - - - + +

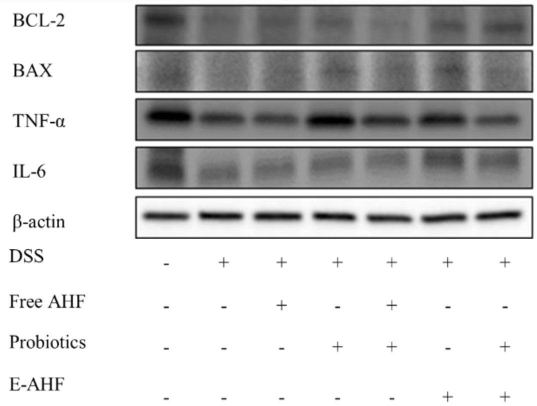
BCL-2
BAX
TNF-α
IL-6
β-actin
DSS - + + + + + +
Free AHF - - + - + - -
Probiotics - - - + + - +
E-AHF - - - - - + +

Figure S3: Effect of dietary supplementations on the expression of tight junction proteins in BALB/c mice colon tissues. (A) Occludin, (B) Claudin-2, (C) Caudin-3, and (D) Claudin-4.

Protein expression was determined using western blot analysis and images were analyzed using Image lab version 6.0.1 software. Results were normalized using the β-actin level and finally expressed as the relative protein levels compared to the control. At least three western blotting experiments were performed, and the results were expressed with means ± standard deviations. One Way Analysis of Variance was performed ($p < 0.05$) with Tukey's multiple mean comparisons ($\alpha = 0.05$) for mean separation. Abbreviations: Free AHF, non-encapsulated anthocyanin rich haskap fraction, E-AHF, encapsulated anthocyanin rich haskap fraction.
